# Investigation of fragment separation during a circular saw blade cutting rock based on ANSYS/LS-DYNA

**DOI:** 10.1038/s41598-022-22267-0

**Published:** 2022-10-16

**Authors:** Zhiwen Wang, Qingliang Zeng, Lirong Wan, Zhenguo Lu, Jun Zhou

**Affiliations:** 1 College of Mechanical and Electronic Engineering, Shandong University of Science and Technology, Qingdao, 266590 China; 2 Shandong Normal University, Jinan, 250358 China; 3 College of Transportation, Shandong University of Science and Technology, Qingdao, 266590 China

**Keywords:** Civil engineering, Energy infrastructure, Mechanical engineering

## Abstract

Circular saw blades are widely used in stone processing. The circular saw blade cutting hard rock numerical simulation model based on ANSYS/LS-DYNA was established to investigate the complex dynamic problem in rock cutting. The failure mechanism of the rock and the influence of cutting parameters on the cutting force and rock fragments were studied by numerical simulation. The results demonstrated that the failure modes of the rock were mainly tensile failure with some shear failure and compressive failure. The cutting force and the number of fragments increased with the feed speed. With the increasing circular saw blade rotational speed, the cutting force and the number of fragments decreased and tended to stabilize. With the distance between the circular saw blades increasing, the cutting force and rock fragments number increase and then maintain basic stability, and when the distance between double circular saw blades reaches 25 mm, it will form a completed rock plate and the interaction of circular saw blades will decrease. The numerical simulation can accurately simulate rock breakage and force when a circular saw blade cuts rock.

## Introduction

Circular saw blades are frequently used in many industries, such as those that cut hard rock, concrete and glass. For hard rock formations, the method of rock breaking and the cutting force during the rock cutting process with circular saw blades are essential for researchers designing circular saw blades and other cutting tools.

Many researchers have conducted many theoretical studies and experimental trials and adopted various numerical simulation methods to consider the cutting force in the process of cutting rock with a circular saw blade. Xu et al. carried out a series of experiments to study the cutting characteristics and the force ratio in circular sawing^1^ and investigated the energy and forces of circular saw blade grinding of granite^2^. Huang et al. proposed a predictive model of sawing power based on tangential force^3^. Aslantas et al. researched the effect of axial cutting force on a circular saw blade used for cutting marble^4^. Karakurt applied the Taguchi method to determine a circular saw blade's cutting force and operational variables^5^. Specific cutting energy is a critical evaluation index of circular saw blade cutting performance. Aydin et al. used an experiment to research the influence of the operating variables and rock properties on specific energy^6^. Yurdakul et al. studied specific cutting energy prediction by statistical methods^7^. Ersoy et al. investigated the effects of rock parameters on the cutting property of circular saw blades with different feed rates and cutting depths^8^. Kahraman et al. established models that used to evaluate slab production and rock properties with a series of performance measurements of large-diameter circular saws^9^.

Many scholars have studied the sawability, damage, rock fragments and wear of circular saw blades. Güney established a model to predict the performance of a large-diameter saw based on rock surface hardness, which can be used to predict the sawability of carbonate^10^. Fener et al. used single and multiple regression analysis to research the correlations between sawability and rock properties ^11^. Ersoy et al.^12^ and Aydin et al.^13^ researched the influence of operating parameters and the characteristics of the cut rock on the wear of a circular saw. Zeng et al.^14^ studied the influence of cutting parameters on coal and rock damage with circular saw blade cutting. Tang et al.^15^ and Liu et al.^16^ researched rock damage based on a statistical rock damage constitutive model. The fragments of rock were studied by Lu et al. based on LS-DYNA^17^. Aydin et al.^18^ investigated the saw blade performance prediction based on the artificial neural network and regression analysis. Tumac et al. had studied the cutting performance with the large diameter circular saw blade^19^ and the prediction of the large diameter sawability performance^20^. Turchetta et al.^21^ studied the cutting force and wear of the circular saw blade under high-speed. Wang et al.^22^ investigated the circular saw blade cutting rock with numerical simulation method. However, the paper studied the cutting parameters of the saw blade influence on rock damage and cutting force, in the process of circular saw blade cutting rock with constant cutting depth. But there is less research about the rock fragment with circular saw blade cutting, and they have investigated the cutting parameters of the flexible saw blade influence on the saw blade deformation and rock damage without the rock fragment in the process of circular saw blade cutting into rock vertically^23^. Lu et al.^24^ have investigated the conical pick breaking rock plate which is formed by the circular saw blade cutting rock, but there is no investigation about the circular saw blade cutting rock. Tao et al.^25^ studied the circular saw blade cutting stone with the nonlinear dynamics finite element simulation method, the paper studied the movement rule and wear mechanism. Wicaksana et al.^26^ have investigated the pick cutter breaking rock in cutting process with numerical simulation method considering rock dynamic properties, in the paper, the investigation is considering dynamic properties of rock.

Previous studies have attained many advancements in circular saw blades. Most researchers have studied the cutting force, specific cutting energy, rock sawability and circular saw blade wear in the process of circular saw blade cutting rock. And there are many researchers researching breaking rock (stone) with other cutter. There is less investigation about the circular saw blade cutting rock forming many fragments and the rock damage. Therefore, the manuscript has investigated the cutting parameters of circular saw blade cutting into rock vertically influence on rock fragments and rock damage with numerical simulation method. And the numerical simulation is modified with the circular saw blade cutting rock experiment. The feed speeds of the circular saw blade are set as 0.10, 0.12, 0.14, 0.16, 0.18, 0.20, 0.22, 0.24, 0.26, 0.28 and 0.30 m/min, the rotational speeds are set as 1000, 1400, 1800, 2200, 2600 and 3000 r/min, and the distance of double circular saw blades are set as 10, 15, 20, 25, 30, 35, 40, 45, and 50 mm, to investigate the influence of cutting parameters on circular saw blade cutting performance and the rock fragments in the process of circular saw cutting rock. Therefore, the simulation results could guide rock processing.

## Methods

### The mathematical expression of the cutting force

**Figure 1:**
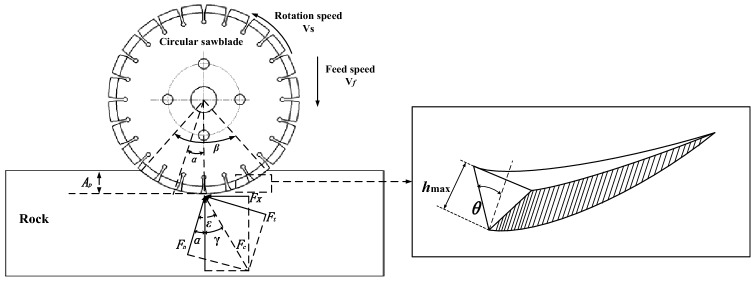
The circular saw blade cutting hard rock model.

In the circular saw blade cutting rock process, the cutting force is composed of the normal force, tangential force, and axial force, as shown in Fig. 1. The normal force is formed by the compression between the circular saw blade and rock. The relative sliding friction between the saw blade and the rock forms the tangential force. The axial force is formed by extruding fragments between circular saw blade and rock wall. And the effect of the diamond abrasive particles of a diamond saw blade on the cutting force during rock cutting is shown in Fig. 1. Comparing with the cutting force, normal force and the tangential force, the axial force is too small to simplify the solving model. Therefore, the equations for solving the tangential and normal forces during the circular saw blade cutting rock are shown in formulas (1) and (2)^27^.

1 $${F}_{t}={F}_{x}{\text{cos}}\left[2k\,{\text{cos}}^{-1}\left(1-\frac{{2A}_{p}}{D}\right)\right]+{F}_{y}{\text{sin}}\left[2k\,{\text{cos}}^{-1}\left(1-\frac{{2A}_{p}}{D}\right)\right],$$

2 $${F}_{n}=-{F}_{x}{\text{sin}}\left[2k\,{\text{cos}}^{-1}\left(1-\frac{{2A}_{p}}{D}\right)\right]+{F}_{y}{\text{cos}}\left[2k\,{\text{cos}}^{-1}\left(1-\frac{{2A}_{p}}{D}\right)\right],$$

3 $$\updelta =\frac{{l}_{s}}{{l}_{s}+{l}_{w}},$$

where ${l}_{s}$ is the arc length of a single segment, ${l}_{w}$ is the arc length of a single flume, $\updelta$ is the continuous ratio of the saw blade, and $\delta =$ 0.713.

4 $${A}_{a}=\frac{C\eta\updelta }{{l}_{s}b},$$

where C is the number of effective abrasives on a single block, ${A}_{a}$ is the number of diamond abrasives cutting per unit area, $\eta$ is the ratio of the effective abrasive particles to the diamond abrasive particles actually involved in cutting and generally is 2/3, and b is the section width of the circular saw blade.

5 $${l}_{c}=\frac{D}{2}\beta ,$$

where ${l}_{c}$ is the sawing arc length, D is the blade diameter, and $\beta$ is the sawing arc angle.

6 $$\beta =2\,{\text{cos}}^{-1}\left(1-\frac{{A}_{p}}{R}\right)=2\,{\text{cos}}^{-1}\left(1-\frac{{2C}_{p}}{D}\right),$$

where ${C}_{p}$ is the single diamond abrasive cutting depth.

The maximum cutting depth of a single diamond abrasive is presented as formula (7),

7 $${h}_{max}=\sqrt{\frac{{V}_{f}}{{V}_{r}}\frac{3}{{A}_{a}\,{\text{tan}}\theta }\sqrt{\frac{{A}_{p}}{D}}},$$

where $\theta$ is half of the angle at the cutting bottom and is taken as 60 degrees, ${V}_{f}$ is the feed speed, and ${V}_{r}$ is the rotation speed.

According to the Fig. 1, the solutions of tangential and normal forces can be expressed as follows:

8 $${F}_{t}={F}_{x}{\text{cos}}\alpha +{F}_{y}{\text{sin}}\alpha ,$$

9 $${F}_{n}=-{F}_{x}{\text{sin}}\alpha +{F}_{y}{\text{cos}}\alpha ,$$

10 $$\alpha =\varepsilon -\gamma ,$$

11 $$\alpha =k\beta ,$$

where ${F}_{t}$ is the tangential force, ${F}_{n}$ is the normal force, ${F}_{x}$ is the horizontal force, and ${F}_{y}$ is the vertical force. Among them, the contact arc length of the circular saw blade and the rock determined the value of $k$; however, $k$ is a dynamic variable.

### The rock constitutive model

The rock is a quasi-brittle heterogeneous material. The RHT constitutive model is applied to simulate the rock, which can be used to research rock damage with circular saw blade cutting. The RHT constitutive model can simulate the rock performance, which has 34 parameter and most parameters can be tested and calculated accurately. The RHT model can be applied to describe the failure strength, initial yield strength and residual strength of rock with load. The RHT model includes three cap surfaces, residual strength surface, failure surface and elastic limit surface, which helps the constitutive model to express the relationship between the hydrostatic pressure, failure strength and elastic limit.

Elastic limit surface function equation:

12 $${Y}_{cla}^{*}={Y}_{fail}^{*}{F}_{CAP}\left(P\right).$$

While the cap of the yield surface is represented by

13 $$ F_{CAP} (P) = \left\{ {\begin{array}{*{20}l} {1,} \hfill & {{\text{for}}\;P^{*} \le \frac{1}{3},} \hfill \\ {1 - \left( {\frac{{P^{*} - \frac{1}{3}}}{{\frac{{P_{0} }}{f} - \frac{1}{3}}}} \right)^{2} ,} \hfill & {\frac{1}{3} < P^{*} < \frac{{P_{0} }}{{f_{c} }}} \hfill \\ {0,} \hfill & {{\text{for}}\;P^{*} \ge \frac{{P_{0} }}{{f_{c} }}} \hfill \\ \end{array} } \right., $$

where ${P}_{0}$ is the elastic limit of the material; ${Y}_{cla}^{*}$ is the ratio of the elastic strength to the ultimate strength of the material; ${P}^{*}$ is the normal hydrostatic pressure.

The corresponding *g* failure function ${Y}_{fail}^{*}$ of the failure surface is expressed as follows:

14 $${Y}_{fail}^{*}={\sigma }_{eq}^{*}\left(P,\theta ,\dot{\varepsilon }\right)={Y}_{txc}^{*}(P){R}_{3}(\theta ){F}_{RATE}(\dot{\varepsilon ),}$$

where ${\sigma }_{eq}^{*}$ is the normalized equivalent stress and normalized as

15 $${\sigma }_{eq}^{*}={\sigma }_{eq}/{f}_{c},$$

where, ${f}_{c}$ is the uniaxial compressive strength, ${Y}_{txc}^{*}(P)$ is the strength of the compressed meridian. The evolution of this variable is given as

16 $${Y}_{txc}^{*}\left(P\right)=A{\left[{P}^{*}-{P}_{SPAIL}^{*}{F}_{RATE}(\dot{\varepsilon )}\right]}^{N},$$

17 $${P}_{SPAIL}^{*}={P}_{SPAIL}/{f}_{c},$$

where ${F}_{RATE}(\dot{\varepsilon )}$ is the dynamic strain rate enhancement factor, shown as the following equation.

18 $$F_{{RATE}} \left( {\dot{\varepsilon }} \right) = \left\{ {\begin{array}{*{20}c}    {\left( {\dot{\varepsilon }/\dot{\varepsilon }_{0} } \right)^{\alpha } ,P > f_{c} /3,\;\dot{\varepsilon }_{0}  = 30 \times 10^{{ - 6}} {\text{ S}}^{{ - 1}} }  \\    {\left( {\dot{\varepsilon }/\dot{\varepsilon }_{0} } \right)^{\alpha } ,P < f_{c} /3,\;\dot{\varepsilon }_{0}  = 3 \times 10^{{ - 6}} {\text{ S}}^{{ - 1}} }  \\   \end{array} } \right., $$

where ${R}_{3}(\theta )$ is the radial radius corresponding to any stress angle and the radial meridional radius ratio, shown as the following equation.

19 $${R}_{3}\left(\theta \right)=(2\left(1-{Q}_{2}^{2}\right){\text{cos}}\theta +\frac{\left(2{Q}_{2}-1\right)\sqrt{4\left(1-{Q}_{2}^{2}\right)\cdot {\text{cos}}^{2}\uptheta +5{Q}_{2}^{2}-4{Q}_{2}}}{4\left(1-{Q}_{2}^{2}\right){\text{cos}}^{2}\theta +{\left(1-2{Q}_{2}\right)}^{2}},$$

in which, $$\theta ={\text{cos}}^{-1}\left(\frac{3\sqrt{3}{J}_{3}}{2{J}_{2}^\frac{3}{2}}\right)/3$$, $$0\le \theta \le \pi /3,$$

20 $${Q}_{2}= {r}_{t}/{r}_{c} = {Q}_{0}+{B}_{Q}{P}^{*},$$

which, 0.51 $\le$ *Q*_2_ $\le$ 1.0, which *A*, *N*, $\partial , \delta , {Q}_{2}$ and ${B}_{Q}$ are the material parameters.

The residual strength surface is described below,

21 $${Y}_{resodual}^{*}=B\times {({P}^{*})}^{M},$$

where, *B* is the residual failure surface constant; and *M* is the residual failure surface index.

The front description is as follows: If the front is located between the elastic limit and the maximum failure surface, then

22 $${Y}_{\text{pre}}={Y}_{ela}+{\varepsilon }_{pl,eq}({Y}_{fail}-{Y}_{ela})/{\varepsilon }_{plhard/eq},$$

where, ${\varepsilon }_{pl,eq}$ and ${\varepsilon }_{plhard/eq}$ are the plastic strain corresponding to the current failure surface and the maximum failure, respectively.

When the front is located between the maximum failure surface and the residual failure surface, the failure surface depends on the amount of damage *D*.

23 $${Y}_{\text{pre}}={Y}_{\text{fail}}-D\left({Y}_{fail}-{Y}_{residual}\right),$$

24 $$D=\sum \frac{\Delta {\varepsilon }_{P}}{{\varepsilon }_{P,failure}}=\sum \frac{\Delta {\varepsilon }_{P}}{{D}_{1}{({P}^{*}-{P}_{spall}^{*})}^{{D}_{2}}}={\int }_{0}^{{\varepsilon }_{P}}\frac{d{\varepsilon }_{P}}{{D}_{1}{({P}^{*}-{P}_{spall}^{*})}^{{D}_{2}}}, {\varepsilon }_{P,failure}\ge {\varepsilon }_{f,min},$$

where, ${\varepsilon }_{f,min}$ is the minimum plastic strain at the time of material failure, taking 0.01, ${D}_{1}$ and ${D}_{2}$ are the material damage constants; ${\varepsilon }_{P}$ is the plastic strain.

### The rock damage model

The cracks inhomogeneous propagation in the space causes the derived materials anisotropy, and the anisotropic behavior needs to be considered to build the constitutive damage relation with the external load action. Each crack is given a damage value to describe the crack state, with considering the nork vector discreteness. The fracture families damage variables are defined as a set, which is expressed as formula (25), ${d}_{i}$ is the damage internal variable of the *i*-th crack.

25 $$D=\left\{{d}_{1}, {d}_{2},\dots ,{d}_{N}\right\}.$$

It should be considered that the rock matrix contains a single group of fractures firstly to simplify. Assuming that the normal vector of the group fractures is ***n***, and the $d=d({\varvec{n}})$, used to represent the fracture distribution density, that is, the damage variable in generally. It is necessary to determine the fracture matrix system free energy expression to build a damage mechanics model based on thermodynamics. Only considering the energy dissipation of crack propagation, the strain-free energy of the system is the function of the macroscopic variable $\varepsilon$ and the damage variable *d*, as plotted in function (26)

26 $$\Psi \left(\epsilon ,d\right)=\frac{1}{2}\epsilon :{C}^{hom}\left(d\right):\epsilon ,$$

where, ${C}^{hom}\left(d\right)$ is the damaged material effective elastic tensor.

Which can be obtained by deriving the free energy from the internal variables. Firstly, establish the macro stress–strain relation as shown in formula (27),

27 $$\sigma =\frac{\partial \Psi }{\partial d}={C}^{hom}:\epsilon .$$

The thermal force related to the damage variable is obtained, which is, the damage drive force.

28 $${F}_{d}=-\frac{\partial \Psi }{\partial d}=-\frac{1}{2}\epsilon :\frac{\partial {C}^{hom}}{\partial d}:\epsilon .$$

According to the second thermodynamics law, the energy dissipation caused by fracture propagation is nonnegative, and it satisfies the Eq. (29)

29 $${D}_{e}={F}_{d}\dot{d}\ge 0.$$

In the framework of the thermodynamics, the damage criterion based on the strain energy release rate is usually adopted, shown as Eq. (30)

30 $$\text{g}\left({F}_{d},d\right)={F}_{d}-R\left(d\right)\le 0,$$

where, $R\left(d\right)$ is the resistance function of damage evolution (crack propagation), For the damage criterion (31). The loading conditions are as follow:

31 $$ \left\{ {\begin{array}{*{20}l}    {{\text{when}}\;F_{d}  < R\left( d \right),\,\dot{d} = 0,\,({\text{fracture without expandion}})}  \\    {{\text{when}}\;F_{d}  = R\left( d \right),\,\dot{d} > 0,\,\left( {{\text{fracture expandion}}} \right)}  \\   \end{array} } \right. $$

Assuming that the rock is orthorhombic material, the damage evolution obeys the orthogonalization criterion.

32 $$\dot{d}={\lambda }^{d}\frac{\partial \left({F}_{d},d\right)}{\partial {F}_{d}}={\lambda }^{d}, {\lambda }^{d}\ge 0,$$

where, ${\lambda }^{d}$ is the damage multiplier.

Similar to the classical plastic theory, the damage evolution equation considering loading and unloading condition is as follow,

33 $$ \dot{d} = \left\{ {\begin{array}{*{20}l}    {0,\;{\text{g}} < 0\,{\text{or}}\,g = 0\,{\text{and}}\;{\dot{\text{g}}} < {\text{0}}}  \\    {\lambda ^{d} ,\;{\text{g}} = 0\, {\text{and}}\,\dot{g} = 0}  \\   \end{array} } \right. $$

The damage multiplier ${\lambda }^{d}$ can be determined by the damage consistency condition (g = 0 and $\dot{\text{g}}=0$), as follow,

34 $$\dot{\text{g}}=\frac{\partial g}{\partial \varepsilon }\dot{\varepsilon }+\frac{\partial g}{\partial d}\dot{d}=0.$$

In addition, the stress–strain relationship in the form of rate can be established based on the damage evolution criterion. Firstly, the macroscopic stress–strain relationship is expressed in differential form as the Eq. (35)

35 $$\dot{\sigma }={\dot{C}}^{hom}:\varepsilon +{C}^{hom}:\dot{\varepsilon }.$$

And there is the following relation, shown as (36)

36 $${C}^{hom}:\varepsilon =\frac{\partial {C}^{hom}:\upvarepsilon }{\partial d}\dot{d}=-\frac{\partial {F}_{d}}{\partial \varepsilon }{\lambda }^{d}=-\frac{\partial g}{\partial \varepsilon }{\lambda }^{d}.$$

And then get the equation as (37)

37 $$\dot{\sigma }={C}^{tan}:\dot{\varepsilon },$$

where, ${C}^{tan}$ is the tangential elastic tensor of the material, the specific expression as (38)

38 $${C}^{tan}={C}^{hom}-\frac{1}{{H}_{d}}\frac{\partial g}{\partial \varepsilon }\otimes \frac{\partial g}{\partial \varepsilon },$$

among them, the damage hardening parameters ${H}_{d}=-\partial g/\partial d$.

### The geometric model and boundaries

**Figure 2:**
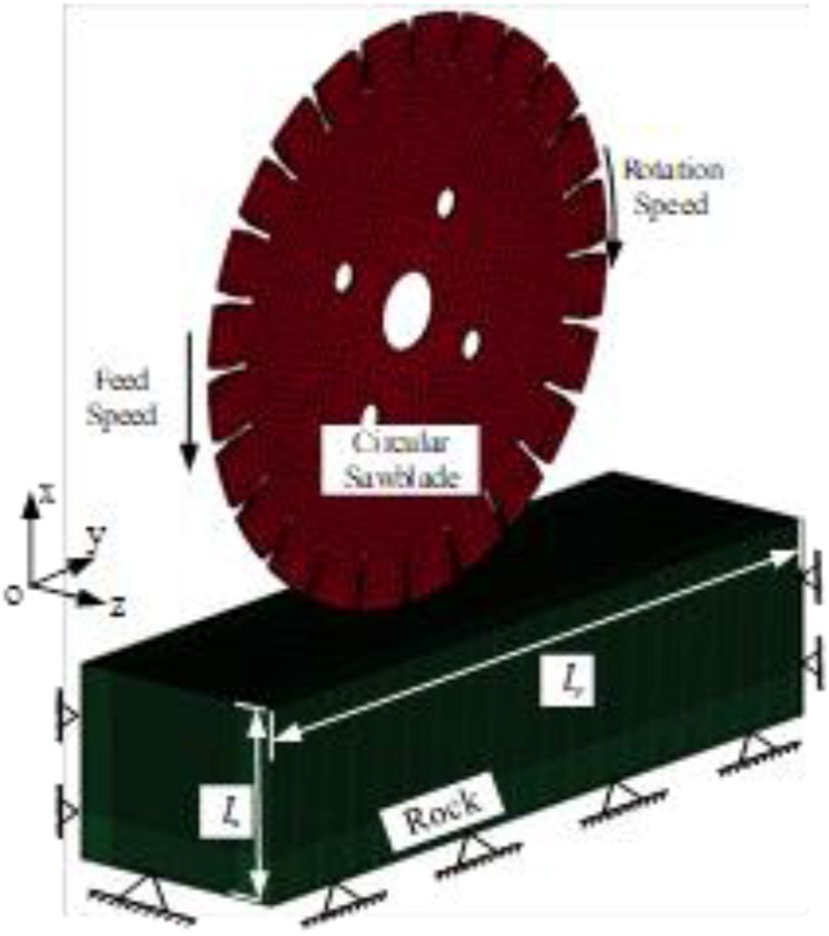
The circular saw blade cutting rock numerical simulation model.

A three-dimensional model of cutting rock with a circular saw blade is shown in Fig. 2. The rock and circular saw blade were simplified as an 800 $\times$ 300 $\times$ 300 mm cuboid, and the circular saw blade model has a 600 mm diameter and 5 mm thickness with 24 U-shaped teeth. The circular saw blade was brazed to the periphery of a circular steel core. The material model RHT and the 8 node-hexahedral SOLID_164 are applied to mesh the rock model, element size is set as 1 mm by the MeshTool. The paper mainly studies the rock fragments and cutting force of the circular saw blade, the circular saw blade is meshed with RIGID material. The key parameters of rock are shown as Table 1. The key material parameters of rock constitutive model and the saw blade model are applied in the numerical simulation model, presented in Tables 2 and 3. The whole constraint was added to the bottom surface of the rock, restricting the displacement in the x, y, z directions (to the right and left of the surface and in front and behind of the surface). The non-reflection boundaries, which are not cut, were added to the rock’s surface to simulate the actual rocks accurately. Aim to solve the interaction problem between the circular saw blade and the rock, the automatic Lagrange/Lagrange coupling was applied^28^. The y and z-directions displacement constraints and the x-axis and y-axis rotation constraints were used for the circular saw blade. The linear motion in the y-direction and rotation around the x-axis were applied to the circular saw blade. The time step was set as 0.01 s to output calculation file. The k file was imported into ANSYS/Slover for calculation with the workstation, 40 computing cores. This paper investigated the rock fragmentation mechanism with circular saw blades during the rock cutting process.

**Table 1:** The key parameters of rock properties.

Density(kg/m^3^)	Compressive strength (MPa)	Tensile strength (MPa)	Poisson’s ratio
2564.76	108.45	6.67	0.209

**Table 2:** The key material parameters of rock properties in the numerical simulation.

Parameter symbols	Value	Parameter symbols	Value	Parameter symbols	Value	Parameter symbols	Value
$\rho$	2670 kg/m^3^	$\beta$_c_	0.0107	*A*_3_	9.04e10	*T*_2_	0
*f*_*c*_	1.0848e8	$\beta$_t_	0.0157	*B*_0_	1.22	*G*	2.51e10
$\alpha$_0_	1.18	*A*_1_	3.57e10	*B*_1_	1.22	${\dot{\varepsilon }}_{0}^{c}$	3.0e−5
*p*_*el*_	3.4e7	*A*_2_	1.64e11	*T*_1_	8.71e9	${\dot{\varepsilon }}_{0}^{t}$	3.0e−6
${\dot{\varepsilon }}^{c}$	3e19	*D*_1_	0.04	*D*_2_	1	${g}_{t}^{*}$	0.7
*A*	1.6	${\dot{\varepsilon }}^{t}$	3e19	*n*	0.61	${f}_{t}^{*}$	0.18
${f}_{s}^{*}$	0.13	*B*	0.0105	${\varepsilon }_{p}^{m}$	0.01	*p*_*comp*_	6e8
*N*	3.0	*A*_*f*_	1.6	*Q*_0_	0.6805	*NF*	0.61

**Table 3:** The key material parameters of circular saw blade properties in the numerical simulation.

Parameter	Value	Parameter	Value	Parameter	Value
Density	7800 kg/m^3^	Shear modulus	2.1e12 Pa	Poisson’s ratio	0.3

### Modification of the numerical simulation

**Figure 3:**
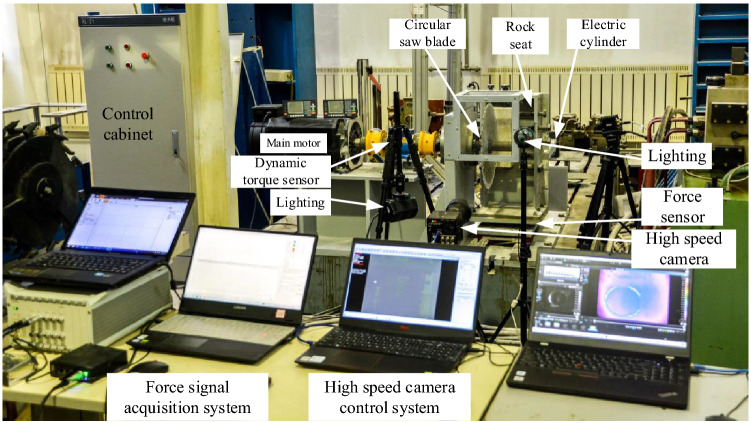
The circular saw blade cutting rock test bench.

Established the circular saw blade cutting rock test bench to conduct the circular saw blade cutting into rock vertically. The circular saw blade cutting rock test bench is shown as Fig. 3. The high-speed camera is applied to photograph the circular saw blade cutting rock, the force sensor and the force signal acquisition system are applied to collect the circular saw blade cutting rock force. The circular saw blade cuts into rock vertically, with rotational speed of 1000 r/min and feed speed of 0.20 m/min.

**Figure 4:**
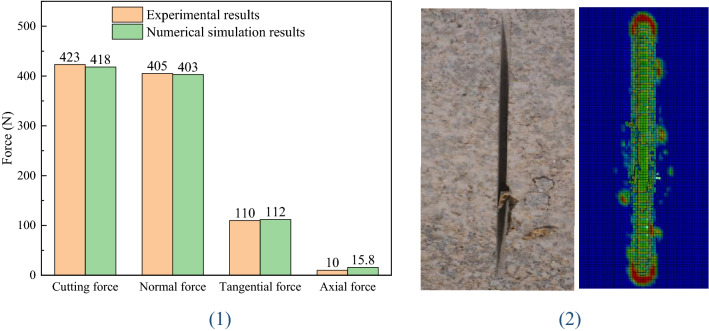
The experimental results of circular saw blade cutting rock.

The experimental results of circular saw blade cutting into rock vertically, are presented in Fig. 4 (1). The cutting force, normal force, tangential force and axial force of the experimental and numerical simulation results error is less than 0.05, therefore the numerical simulation is accurate. And the results of the experiment and numerical simulation are shown in Fig. 4 (2), the results of the numerical simulation indicated that the rock breaking forming several fragments with the circular saw blade cutting. And the results of experiment shown that there are several rock fragments. The results of the circular saw blade cutting rock experiment modified the numerical simulation which helps to improve the accuracy of numerical simulation.

## Results and discussion

### The process of dynamic rock fragmentation

**Figure 5:**
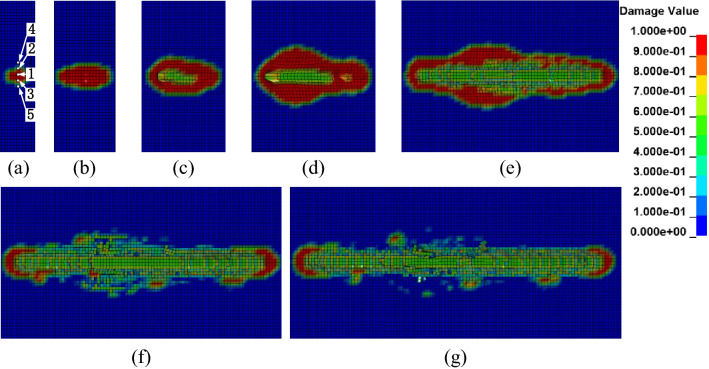
The circular saw blade cutting rock with a cutting depth of 10 mm, feed speed of 0.3 m/min, rotational speed of 3000 r/min.

The numerical simulation results of the rock fragmentation process when cutting rock with a circular saw blade at an advanced speed of 0.3 m/min and a rotational speed of 3000 r/min are shown in Fig. 5. In the process of rock cutting, the circular saw blade compresses the rock, the damage field of the rock formed and propagated in the initial stage, and then the damage zone expanded. The damage region of rock was generated at the contact position around the circular saw blade, as depicted in Fig. 5a. The elements behind the circular saw blade were first affected by the saw blade, which resulted in damage and strain. The obvious strain appeared in the front center of the contact area of the circular saw blade and rock, and closer to the front of the centre of the contact position of the circular saw blade and rock failure, the rock element strain was distinct, with the edge of the saw blade segment exerting the cutting force on the rock elements, as shown in Fig. 5b. Most of the elements on both sides of the circular saw blade did not fail primarily because the rock generated elastic deformation. As shown in Fig. 5c, the damage field extended with the continuation of the rock cutting, and some elements in the damage field failed. The rock element deformation occurred at the position of the circular saw blade cutting rock. While the saw blade rotated, the element’s deformation contacting the edge of the saw blade segment was obvious. As the cutting depth increased, the contact area of the circular saw blade and the rock increased, and the damage field length increased; however, the width of the damage field decreased. The number of failure elements increased as the circular saw blade cut rock, plotted in Fig. 5d. The damage area decreased with the circular saw blade cutting rock, and the rock broke up to form fragments, as shown in Fig. 5e. By comparing Fig. 5d,e, it is apparent that the damage field on both sides of the circular saw blade decreased and the number of failure elements increased.

Some fragments formed as the cutting depth increased and the damage area decreased. Some elements in the damage field did not fail, primarily due to the rock-generated elastic deformation and compressive stress. Figure 5f shows that many fragments fell off the rock, and the damage zone decreased. The position of the crack was random owing to the rock is a kind of heterogeneous material, as shown in Fig. 5d–f. As plotted in Fig. 5f, more fragments appear without regularity, forming some fracture regions. In the circular saw blade’s front and rear damage regions, the elements failed to simulate the rock-forming rock fragments as the saw blade cut the rock. The fragments were mainly formed in the middle part of the cutting area, and the larger fragments were separated from the intermediate area of the intersection zone of the circular saw blade and the rock.

Comparing Fig. 5d–f, with the cutting depth increasing, it is apparent that the damage field decreased, the elements failed, and some fragments formed in the rock cutting area. As the cutting depth increased, the damage area declined, and the number of failure elements and fragments increased. Deletion of failure elements caused some elements that did not reach the failure criteria to separate from the rock and form fragments. Most fragments were formed at the middle part of the cutting area on both sides of the circular saw blade. The circular saw blade cut rock with a high rotational speed, and many fragments formed, becoming the medium of the interaction of the saw blade and the rock on both sides of the saw kerf, which was the main reason for the development of the axial force. The force applied on both sides of the saw slot is more significant in the middle of the cutting area, causing serious breakage and a larger damage field.

**Figure 6:**
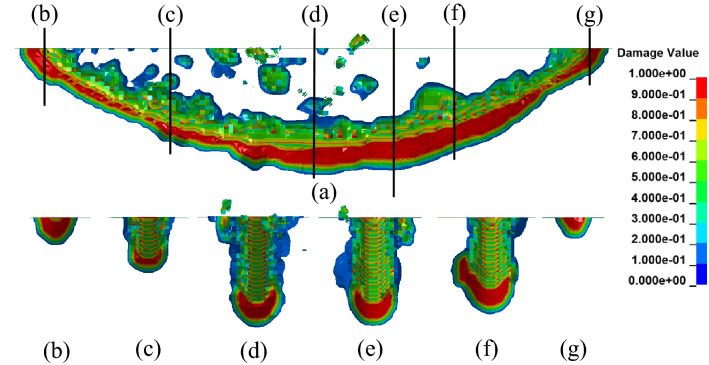
The distribution of different cross-sections in the y-direction.

To research the damage distribution of the rock, different cross-sections were used, as shown in Fig. 6. Six sections in the y-direction and one section in the x-direction indicated that the rock fragmentation and the damage were distributed along the circular saw blade in the rock interior. The main damage zone was distributed along the arc and on both sides of the circular saw blade. The damage field was distributed randomly, and the rock did not fail or form fragments falling off the rock. Comparing the six profiles (Fig. 6b–g) corresponding to Fig. 6a, it can be concluded that the distribution of the damage field along the longitudinal depth of the saw blade is closely related to the rotational speed and feed speed. The damage zone is not uniformly distributed in Fig. 6a; the damage field increases from the left to the middle and decreases from the middle to the right with an anticlockwise rotational speed and downward feed speed. The area with the greatest damage is in the middle, and the left side has the least damage. Due to the effect of the simulated rotational speed and feed speed on rock, the saw blade damage to the rock is enhanced. The damage area on the right side is larger than that on the left side owing to the fragment pair formed by the saw blade cutting. The damage field is distributed randomly at the upper part of the arc edge of the saw seam, and the rock fragments are mainly at the upper surface.

The distributions of the different cross-sections clearly showed the different erosion distances between the two sides of the saw blade and the rock at different depths. The circular saw blade cutting depth has a significant influence on cutting performance. At a larger cutting depth, there are many cracks intersecting and forming too many fragments are caused by the isotropy and continuity of the rock. It is indicated that the numerical simulation of rock cutting with a circular saw blade can reproduce the rock fragmentation process.

### The rock failure mode

**Figure 7:**
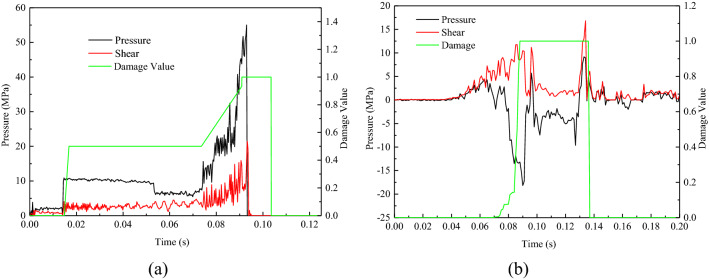
Pressure and corresponding damage versus time in different positions.

To investigate the rock fragmentation mechanism for the interaction between the circular saw blade and rock, five failure elements were selected in the crushing field at the first contacting point between the circular saw blade and rock during the circular saw blade cutting into rock vertically, as presented in Fig. 5, to gauge the stress, pressure and damage value versus time to research the failure in rock cutting with a circular saw blade, as shown in Fig. 7. A positive value of the pressure indicates compressive stress, while a negative value indicates tensile stress. There may be three different kinds failure modes occurring in the circular saw blade cutting rock process, including compressive, tensile and shear failure. The research method is the similar with the reference^22^ and likely to reference^25^. While the element tensile stress reaching 18.2 MPa, indicated tensile failure, the shear stress reaching 39.7 MPa predicted shear failure, otherwise, the element exhibits compressive failure.

As shown in Fig. 7a, the test point 1 (element 124,123) pressure reached 55.1 MPa; the shear stress was 21.5 MPa, which is less than 39.7 MPa, and the tensile stress was less than the tensile strength threshold of 18.2 MPa and the damage value reached 1, which be determined that test point 1 is a compressive failure. The damage value of test point 2 got 1, and the tensile stress reached 18.18 MPa due to a relatively low extraction value; the shear stress was 16.83 MPa, which means that failure did not result from the shear stress, indicating that the test point 2 failure mode was a tensile failure, shown in Fig. 7b. For test point 4, shear stress reached 39.7 MPa; however, the tensile stress and strength stress was less than the tensile strength of 18.2 MPa, which indicated shear failure.

Among the five test points, 3 points exhibited tensile failure, 1 shown compressive failure, and 1 indicated shear failure. So, it can be summarized that the rock elements failure mode is mainly tensile failure; a few are compressive and shear failures, as shown in Table 4.

**Table 4:** The results of the test points.

Test point	1	2	3	4	5
Compressive stress (MPa)	55.1	32.1	23.21	27.18	24.89
Tensile stress (MPa)	− 8.7	− 18.18	− 18.19	− 16.32	− 18.16
Shear stress (MPa)	34.6	29.32	18.76	39.7	15.64
Damage	1	1	1	1	1

### The influence of the feed speed on cutting performance

**Figure 8:**
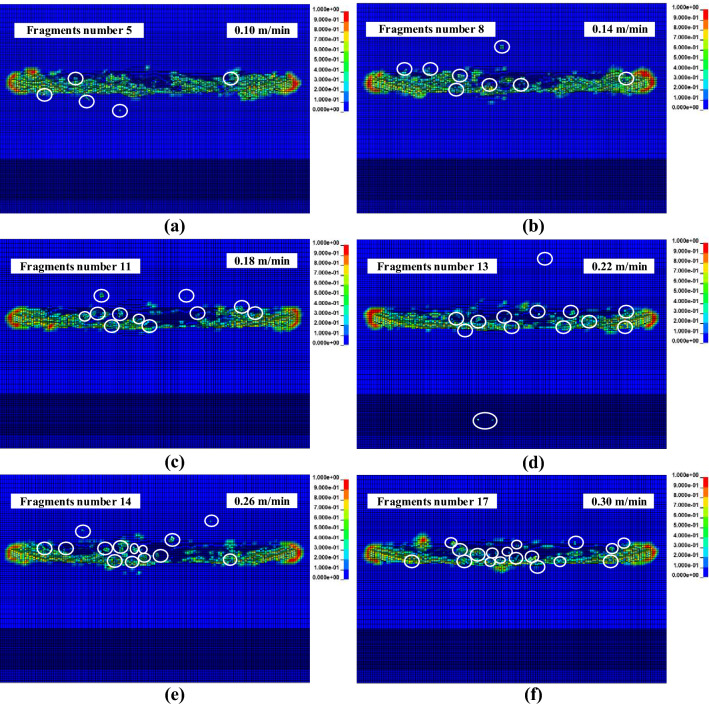
Rock fragmentation with a circular saw blade cutting at various feed speeds.

The circular saw blade cut hard rock with a rotational speed of 2000 r/min and feed speeds of 0.10, 0.12, 0.14, 0.16, 0.18, 0.20, 0.22, 0.24, 0.26, 0.28 and 0.30 m/min. Figure 8 shows the fragments from the circular saw blade cutting rock with feed speeds of 0.10, 0.14, 0.18, 0.22, 0.26 and 0.30 m/min. As the feed speed increased, the number of rock fragments noticeably increased. When the feed speed was 0.10 m/min, the fragments were less, and the extent of the damage area of the rock was indistinct with increasing feed speed. The damaged elements did not fail to decrease. The higher feed speed accelerates the damage of the rock model; when the damage value reaches 1, the element fails and is deleted. When the feed speed was low, the elastic or plastic deformation of the rock element did not reach the failure strength of the rock element; however, when the feed speed was higher, the strain of the rock reached the failure strain, causing the rock element to fail and be deleted. The feed speed greatly affects rock fragment formation, and rock fragments increase with increasing feed speed.

**Figure 9:**
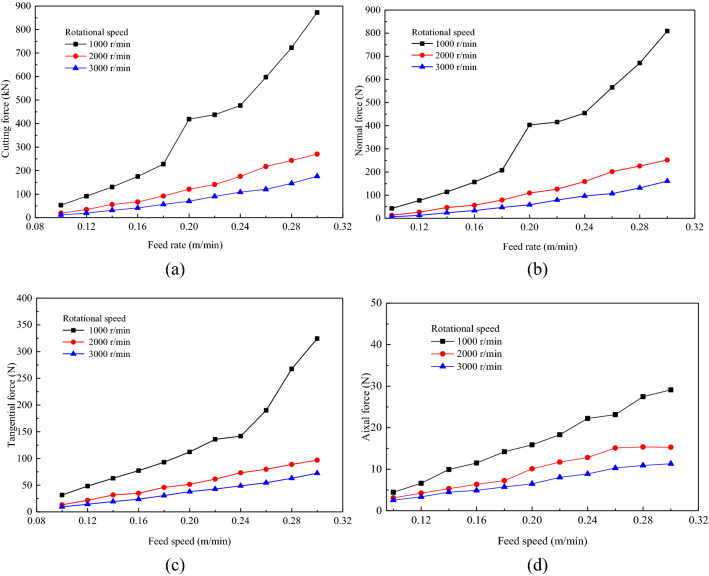
The force responses under different rotational speeds.

Numerical simulations were carried out on a circular saw blade cutting rock at different feed speeds. The force was obtained from the results of the numerical simulation. The cutting, normal, tangential and axial forces were compared with different cutting speeds to investigate the feed speed influence on force. The cutting force increased as feed speed increased, as indicated in Fig. 9a. The normal force curves are indicated in Fig. 9b. And the trend of normal force changing with feed speed is the same as cutting force, and normal force increases with increasing feed speed. The feed speed greatly influences the force, and the cutting force increases with increasing feed speed. The feed speed's tangential force and axial force curves are plotted in Fig. 9c,d, and the tangential force and axial force increase with increasing feed speed, which is coincided to the analysis of Ref.^23^.

### The influence of the rotational speed cutting performance

**Figure 10:**
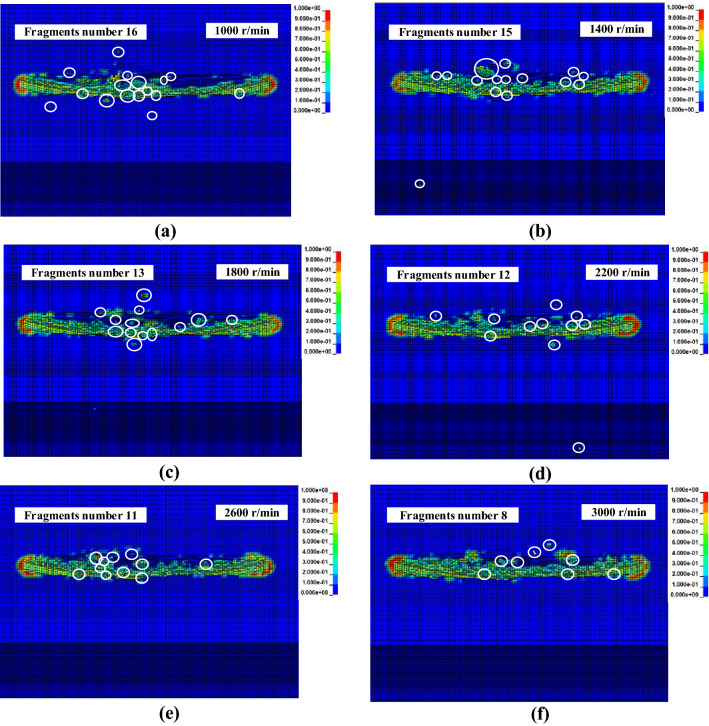
Rock fragments from the circular saw blade cutting at various rotational speeds.

The fragments of the circular saw blade cutting rock at different rotational speeds of 1000, 1400, 1800, 2200, 2600 and 3000 r/min at a feed speed of 0.20 m/min are plotted in Fig. 10. Figure 10 shows that the number of rock fragments decreases with the increase in rotational speed. It is evident that the rotational speed significantly influenced fragment formation and the damage field. The rock damage field change trend is similar with the Ref.^22^, however, the rock fragments with various parameters have not been investigated. The rock fragment is related to rock damage. Therefore, the rock fragments and rock damage should be discussed together. The rock damage area with the circular saw blade decreased with increasing rotational speed, while the number of rock fragments decreased.

**Figure 11:**
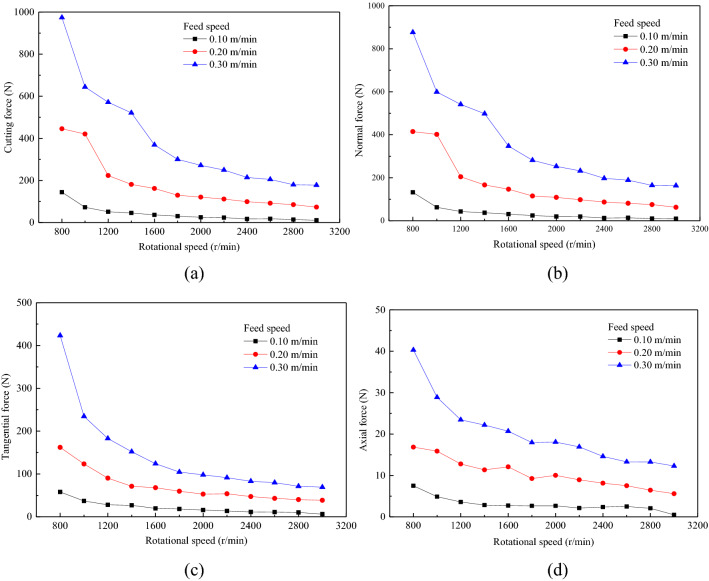
The variation of mean force with rotational speed for different feed speeds.

The influence of the rotational speed on force is shown in Fig. 11. As the rotational speed increased, the force noticeably decreased, and the range of the decrease also reduced. With the increase in the rotational speed, the units of the circular saw blade advanced, the number of rotational cycles increased, the amount of cutting of the hard rock by one process decreased, and the interaction force between the circular saw blade and the hard rock decreased. In addition, the amount and volume of rock fragments decreased, and the amount of cutting per cycle decreased. The rotational speed of the circular saw blade increased, causing the damage zone on both sides of the saw kerf to decrease. With increasing rotational speed, the amount of scraping between the circular saw blade and the rock wall on both sides of the saw slot decreased; the interaction between the saw blade and the rock walls on both sides of the saw slot decreased, causing a decrease in the axial force.

**Figure 12:**
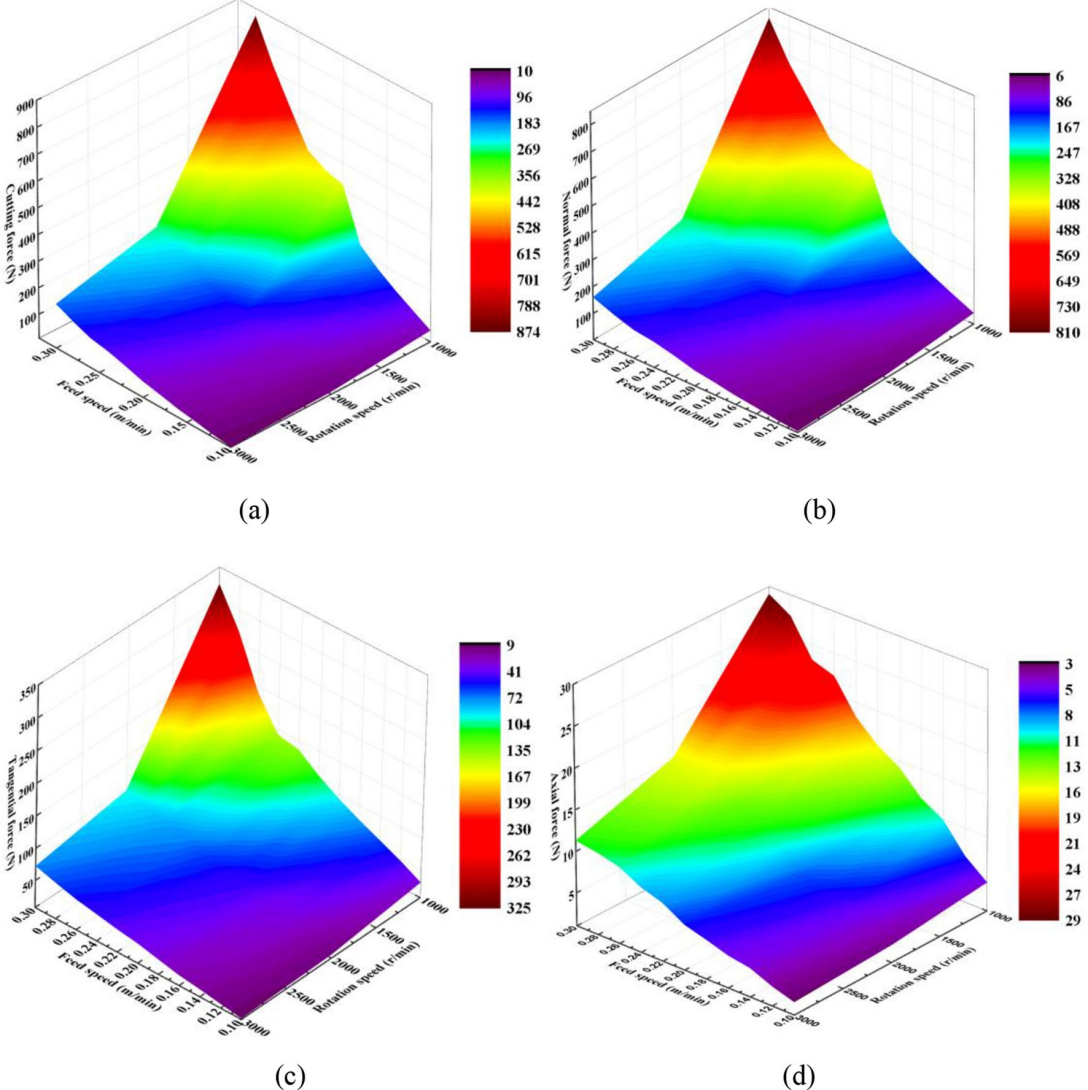
3-D force cloud image of feed velocity and rotation speed.

A three-dimensional force cloud image with different feed and rotational speeds was compared and analysed. The influence of the feed speed on force is more noticeable than that of the rotational speed. The rotational speed decreased as the feed speed increased, causing the force to increase. Figure 12 shows that the cutting force of the circular saw blade increases as feed speed increase and rotational speed decreases. The cutting parameters have a great influence on force. A larger rotational speed and a smaller feed speed are selected to cut hard rock to reduce the force. However, the lower feed speed and greater rotational speed decrease the cutting efficiency.

### The effect of the distance between the double circular saw blades on the cutting performance

**Figure 13:**
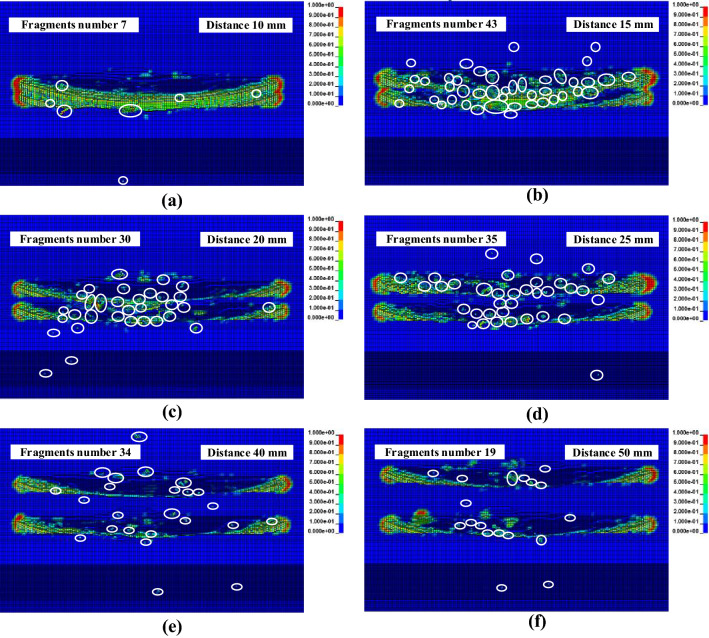
Rock fragments with various spacings between double circular saw blades.

Double circular saw blades cutting hard rock with a feed speed of 0.3 m/min; a rotational speed of 3000 r/min; the spacings between the double saw blades of 10, 15, 20, 25, 30, 35, 40, 45, and 50 mm were compared. The rock fragmentation results are plotted in Fig. 13. When the distance was small, no rock slab was formed between the saw blades; the rock was broken entirely, and the number of rock fragments formed by interaction with the circular saw blade was small. Small spacing between double circular saw blades resulted in the overlapping of the force exerted by the circular saw blades on the rock, which reached the failure strength of the rock and removed the failed rock, forming a larger saw gap. When the spacing between the double saw blade was small, it was difficult to form a complete slab between the double circular saw blades. When the distance between the double saw blades reached 15 mm, a section of rock slab was formed between the double saw blades. Because of the superposition of the saw blade force, the middle part of the rock slab failed and was broken, and there were many rock fragments formed, as shown in Fig. 13b. The amount of rock slab increased when the space between the saw blades reached 20 mm; the rock slab was less broken, and there were fewer rock fragments than when the space between the blades was 15 mm, as depicted in Fig. 13c. As the spacing between the circular saw blades increased, the width of the rock slab formed increased. The circular saw blade cutting rock formed many fragments, but as the distance between the two circular saw blades was small, the number of fragments was less.

**Figure 14:**
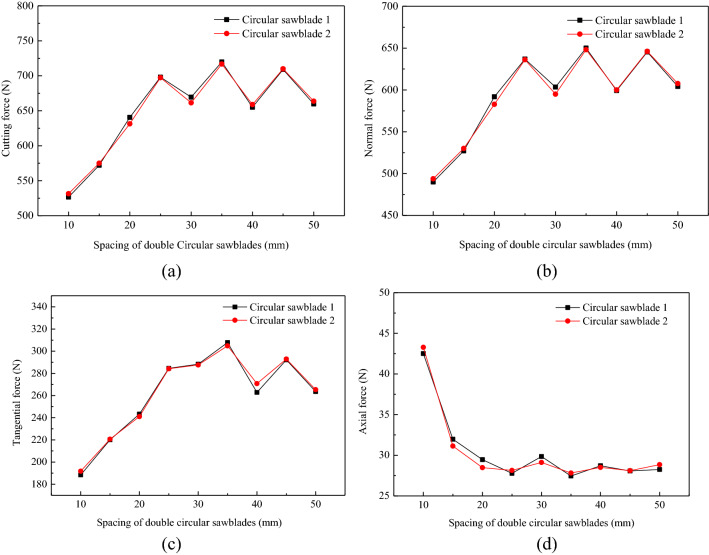
Force of the double circular saw blades with various spacings cutting rock.

The forces of the double circular saw blades with various spacings cutting rock are plotted in Fig. 14. The cutting force increased first and then stabilized with increasing spacing. The normal and tangential force variations with increasing spacing were similar to cutting force. However, the variation in the axial force with saw blade spacing was quite different, as shown in Fig. 14d. The axial force varied, decreasing first and stabilizing with increasing spacing. The influence of the distance on the axial force is obvious, as presented in Fig. 14d. When the space reached 25 mm, the cutting, normal, tangential, and axial forces fluctuated steadily. The forces of circular saw blade 1, and circular saw blade 2 are different. Owing to the rock being an anisotropic material, the force of the saw blades had a slight difference between the two circular saw blades. The double saw blades cut the rock with the same rotational speed and feed speed, and the uncut area between the saw blades was superimposed by the same force field of the saw blade, which reduced the cutting force. However, as the distance of two circular saw blades increased, the stress overlap between the saw blades decreased, and the cutting force, normal force, and tangential force increased. The axial force of the circular saw blades decreased because the axial interaction between the saw blades decreased with increasing separation distance. The axial force is mainly caused by the extrusion of fragments between the rock and saw blade. When the distance between the circular saw blades was over 25 mm, the axial force of the saw blades tended to be stable.

### The cutting parameters on specific cutting energy

**Figure 15:**
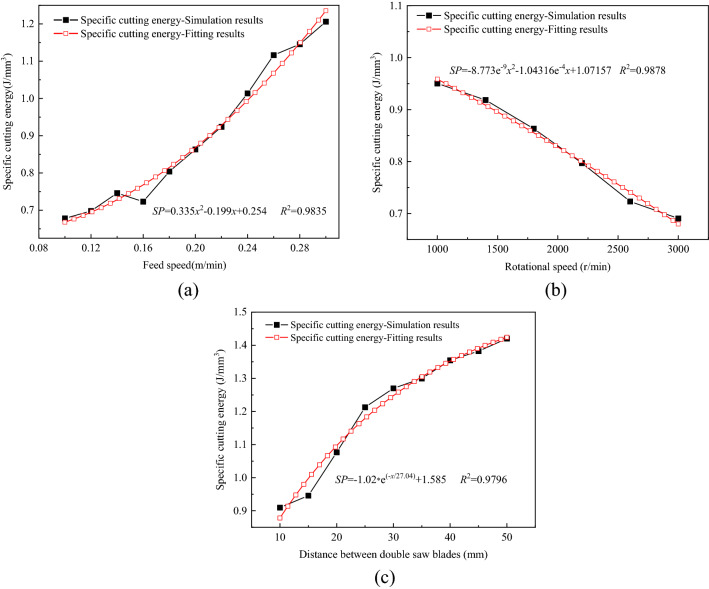
Specific cutting energy of circular saw blade with various cutting parameters.

The specific cutting energy of the circular saw blade cutting into rock is an important index of the cutting performance. And the cutting parameters of circular saw blade have great effect on specific cutting energy. The specific cutting energy of circular saw blade is defined as the ratio of cutting power consumption of saw blade to the sum volume of the broken volume and the rock damage value of 1 volume. The research results of the circular saw blade specific cutting energy. The specific cutting energy curves with various feed speeds, rotational speeds and the distance between double saw blades are shown in Fig. 15.

The feed speed has great influence on specific cutting energy, and the relationship between specific cutting energy consumption and feed speed is quadratic function. With the feed speed increasing, the specific cutting energy increasing, as plotted in Fig. 15a. The cutting force of the circular saw blade increases with the feed speed increasing, which caused the circular saw blade cutting power increasing, however, the rock broken volume has little change. And the *R*^2^ fitting curve function is 09835, and the *P* value is 9.79e−4, indicated the fitting function is accurate and reliable. The specific cutting energy consumption declines with the rotational speed increasing, as shown in Fig. 15b. The *R*^2^ fitting cure function is 0.9878 and the *P* value is 2.24e−4, indicated the fitting function is accurate and reliable. The cutting power consumption declines with the rotational speed increasing, which caused the specific cutting energy declining with the increasing rotational speed. The distance between the double saw blades influences the specific cutting energy of the saw blade greatly. The increasing distance between double saw blades causes the specific cutting energy increasing, however the increasing speed declining, shown in Fig. 15c. Comparing with the specific cutting energy of saw blade cutting rock with various cutting parameters research results in the Ref.^22^, the specific cutting energy has same coinciding variant form.

## Conclusions

This paper established a three-dimensional simulation model about the circular saw blade cutting rock based on ANSYS/LS-DYNA to research the effect of cutting parameters on cutting performance and rock fragment mechanisms during vertical cutting of rock with a circular saw blade.

1. The numerical simulation results indicated that the rock’s failure modes are primarily tensile failure, and a few modes are a shear failure and compression failure.
2. The cutting parameters have an obvious effect on the cutting force and number of rock fragments in circular saw blade cutting rock process. With increasing rotational speed, the cutting force of the circular saw blade and the number of rock fragments decreased and then tended to be stable. However, the cutting force and the number of fragments increased with increasing feed speed.
3. With increasing spacing between double circular saw blades, the cutting force, normal force and tangential force increased, but the axial force decreased. The spacing between the double circular saw blades greatly influenced formation of a slab between the double circular saw blades. As the spacing between the circular saw blades increased, the number of rock fragments increased, and the rock slab tended to be unbroken.
4. The cutting parameters have great influence on specific cutting energy. With the feed speed and distance between double saw blades increasing, the specific cutting energy increase. But the increasing rotational speed causes the specific cutting energy declines.

The investigation results can be used to select the appropriate cutting parameters to guide the rock-cutting process with circular saw blades.

## Data Availability

The data used to support the findings of this study are included within the article.
